# Novel Hematological Parameters in the Assessment of the Extent of Cardiac Implantable Electronic Device-Related Infections

**DOI:** 10.3390/jcm12237498

**Published:** 2023-12-04

**Authors:** Maciej Polewczyk, Wojciech Jacheć, Dorota Szczęśniak-Stańczyk, Anna Polewczyk, Andrzej Tomaszewski, Wojciech Brzozowski, Dorota Nowosielecka, Andrzej Kutarski

**Affiliations:** 1 Institute of Medical Sciences, Jan Kochanowski University, 25-369 Kielce, Poland; maciek.polewczyk@gmail.com; 2 Department of Acute Cardiac Care, Świetokrzyskie Cardiology Center, 25-736 Kielce, Poland; 3 2nd Department of Cardiology, Faculty of Medical Sciences in Zabrze, Medical University of Silesia, 41-800 Zabrze, Poland; wjachec@interia.pl; 4 Department of Cardiology, Medical University of Lublin, 20-059 Lublin, Polandbenecho2008@gmail.com (A.T.); brzozo@wp.pl (W.B.); akutarski@yahoo.com (A.K.); 5 Department of Cardiac Surgery, Świetokrzyskie Cardiology Center, 25-736 Kielce, Poland; 6 Department of Cardiology, The Pope John Paul II Province Hospital, 22-400 Zamość, Poland; dornowos@wp.pl; 7 Department of Cardiac Surgery, The Pope John Paul II Province Hospital, 22-400 Zamość, Poland

**Keywords:** hematological parameters, cardiac implantable electronic device-related infections, extension of CIED-related infections, vegetations

## Abstract

Background: Patients with infectious complications related to the presence of cardiac implantable electronic devices (CIED) constitute a heterogeneous group, ranging from local pocket infection (PI) to lead-related infectious endocarditis (LRIE) infection spreading along the leads to the endocardium. The detection of isolated LRIE and the assessment of the spread of infection in a patient with PI is often difficult and requires complex imaging and microbiological tests. The aim of the current study is to evaluate the usefulness of new simple hematological parameters in detecting infectious complications in patients with CIED, differentiating vegetation and vegetation-like masses, and assessing the extent of infections in patients with PI. Methods: A retrospective analysis of clinical data of 2909 patients (36.37% with CIED-related infections), undergoing transvenous lead extraction (TLE) procedures in three high-volume centres in the years 2006–2020, was conducted. Receiver operating characteristic (ROC) curve analysis was used to assess the sensitivity and specificity of neutrophil-to-lymphocyte ratio (NLR), neutrophil-to-platelet ratio (NPR), and lymphocyte-to-platelet ratio (LPR) in the diagnosis of CIED infections, evaluate the spread of the infectious process in patients with PI and differentiate additional structures related to the presence of lead. Results: The values of NLR and NPR were significantly higher in infectious patients than non-infectious controls (3.07 vs. 2.59; *p* < 0.001, and 0.02 vs. 0.01; *p* = 0.008) and the area under the ROC curve (AUC) was 0.59; *p* < 0.001 and 0.56; *p* < 0.001, respectively. The high specificity of the new markers in detecting the infectious process was demonstrated: 72.82% for NLR (optimal cut-off value: 3.06) and 79.47% for NPR (optimal cut off value: 0.02). The values of NLR and NPR were significantly higher in patients with vegetations than in non-infectious patients with the presence of additional lead-related masses (3.37 vs. 2.61; *p* < 0.001 and 0.03 vs. 0.02; *p* = 0.008). The AUC of NLR and NPR for the prediction of vegetations was 0.65; *p* < 0.001 and 0.60; *p* < 0.001 with the highest specificity of NPR (82.78%) and an optimal cut-off value of 0.03. NLR and NPR were higher in patients with LRIE compared to isolated PI (4.11 vs. 2.56; *p* < 0.001 and 0.03 vs. 0.02; *p* < 0.001) and the ROC curve analysis for coexistence LRIE with PI showed the AUC for NLR: 0.57; *p* < 0.001 and AUC for NPR: 0.55; *p* = 0.001. High specificity in the detection of coexistence between PI and LRIE was demonstrated for NLR (87.33%), with an optimal cut-off value of 3.13. Conclusions: Novel hematological markers (NLR and NPR) are characterized by high specificity in the initial diagnosis of CIED infections, with optimal cut-off values of 3.06 and 0.02. NLR is also useful in the assessment of the spread of infection in patients with PI, with a calculated optimal cut-off value of 3.13. NPR may be helpful in the differentiation of vegetation and vegetation-like masses with an optimal cut-off value of 0.03.

## 1. Introduction

Cardiac implantable electronic device (CIED)-related infections constitute a heterogeneous group of diseases with a poor prognosis. According to recent large studies, in-hospital mortality was 24%, 1-year mortality was 32%, and long-term survival (mean follow-up 5.5 years) was approximately 65% [1,2,3]. The growing number of patients with CIED-related infections is associated with increased hospitalization times and costs and requires more effective strategies to prevent and treat such complications. The spectrum of infectious complications in patients with CIED includes pocket infection (PI) and lead-related infectious endocarditis (LRIE). LRIE may coexist with pocket infection or occur in an isolated form [3,4]. There are two pathophysiological mechanisms of lead-related infectious endocarditis, which include contamination of the leads and/or generator during implantation or subsequent CIED-related procedures, and bloodstream infection, which may occur in the setting of bacteremia due to a distant infection [5]. The diagnosis and the assessment of the extent of an infection in patients with CIED is often difficult. The symptoms might develop slowly with short episodes of fever which are often ignored by both the patient and the doctor [6]. If local signs of pocket infection are present, a proper diagnosis seems to be easier; however, the assessment of the spread of the inflammatory process is still challenging. Similar to other infectious diseases, a gradual increase in the number of neutrophils and a simultaneous decrease in the number of lymphocytes is a typical change in leukocytes in response to acute bacterial infections, while a new hematological parameter—the neutrophil-to-lymphocyte ratio (NLR)—may reflect the severity of the disease [7]. During an infection, we also observe an increase in thrombotic processes associated with increased platelet count. New lymphocyte-to-platelet ratio (LPR) and neutrophil-to-platelet ratio (NPR) parameters may be also helpful in the assessment of the intensity of this phenomenon in patients with LRIE [8]. 

The aim of the current study was to evaluate the sensitivity and the specificity of new simple hematological parameters in the detection of CIED-related infection, confirming/excluding the presence of vegetation, and assisting the assessment of the extent of the infectious process. 

## 2. Methods

### 2.1. Study Group

The retrospective analysis of data of 2909 patients undergoing transvenous lead extraction (TLE) in three high-volume centers in Poland (Zamość, Radom and Lublin) by one main operator in the years 2006–2020 was conducted. All information relating to patients and procedures was entered into the computer on an ongoing basis. The procedures were performed because of both non-infectious and infectious indications. 

Non-infectious indications included various types of lead dysfunction: breaking of the lead; dislocations (loops of the leads); late dry perforations of the exit block type with disorders of pacing, sensing, and resistance; symptomatic venous obstruction; and prophylactic extractions of abandoned, redundant leads. Infectious indications included isolated pocket infection and lead-related infective endocarditis (with or without PI). Additionally, a comparative analysis was performed between a group of patients where vegetation was present (connected with the lead or valve) and a non-infectious group with additional structures on the lead (vegetation-like masses).

### 2.2. Definitions

According to the current guidelines [9], certain LRIE diagnosis was confirmed if two major criteria or one major and three minor criteria were fulfilled. Isolated pocket infection was characterized by the local signs of infection, including swelling, warmth, erythema, and a significant skin erosion around the device pocket [9]. 

All patients underwent preoperative transthoracic echocardiography (TTE) and in all patients with suspected LRIE, transesophageal echocardiography (TEE) was performed. Vegetations and vegetation-like masses were recognized based on echocardiographic examinations. Vegetations were defined as multi-shaped, mobile masses of inhomogeneous echogenicity (hypoechoic in the initial phase of infection) attached to the leads or to the neighboring anatomic structures. They were only found if they were accompanied by signs of a general infection (fever, shiver, positive inflammatory markers, positive blood cultures) or a regional infection (pocket infection) [10,11].

Vegetation-like masses were defined as structures with heterogeneous echogenicity and irregular contour, attached to the leads or surrounding heart structures and varying in size, often being smaller than vegetations. Vegetation-like masses may be the remnant vegetations after antibiotic treatment or organized fibrotic thrombi and their occurrence is only observed in patients without clinical symptoms of infection [10,11,12].

### 2.3. Laboratory Markers

We analyzed laboratory tests of patients qualified for transvenous lead extraction, performed in referral centers and at TLE centers. The hematological results included: lowest values of hemoglobin level, hematocrit level and platelet count, highest value of white blood cell (WBC) count, neutrophil count and ratio, highest value of erythrocyte sedimentation rate (ESR), C-reactive protein (CRP), and procalcitonin. Furthermore, we analyzed neutrophil-to-lymphocyte ratio (NLR), neutrophil-to-platelet ratio (NPR) and lymphocyte-to-platelet ratio (LPR) Laboratory test results were compared in patients with infectious and non-infectious indications for TLE, along with individual groups of infectious patients and patients with vegetation and vegetation-like masses.

### 2.4. Statistical Analysis

Statistical analyses were conducted using Statistica v. 13.3 (TIBCO Software Inc, Palo Alto, CA, USA). Categorical variables were expressed in numbers and percentages, and continuous variables as either the mean and standard deviation (SD) or median, depending on the variable distribution. The variables were compared using the nonparametric Chi2 test with Yates correction (dichotomous data) or the unpaired Mann–Whitney U test (continuous data), as appropriate. Receiver operating characteristic (ROC) curve analysis was used to assess the value of laboratory markers in predicting CIED-related infections, the development of LRIE in patients with PI, and the presence of vegetation. Optimal cut-off levels were determined, and their sensitivities and specificities were calculated. A *p*-value less than 0.05 was considered statistically significant. 

### 2.5. Approval of the Bioethics Committee

The study was conducted according to the ethical guidelines of the Declaration of Helsinki and approved by the Bioethics Committee at the Regional Chamber of Physicians in Lublin no. 288/2018/KB/VII.

## 3. Results

**Figure 1:**
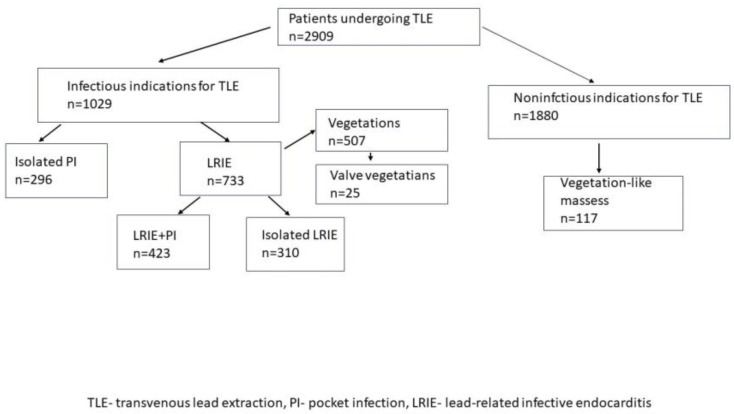
Flowchart of the study population.

The study group consisted of 2909 patients (mean age 69 years; 46.06% female). Infectious indications for TLE occurred in 1029 (36.37%) patients, while TLE for non-infectious reasons was performed in 1880 (64.63%) patients. There were 296 patients (10.18%) with isolated PI and 733 patients (25.20%) with LRIE (with and without PI) in the infectious group. The group of patients with vegetation consisted of 507 patients (17.43%) and the group with vegetation-like masses consisted of 117 patients (4.02%) (Figure 1, Table 1).

The study group consisted of patients with an average age of 69 years, and the mean age at first implantation was 61 years. The study population included 1340 (46.06%) women. The mean left ventricular ejection fraction (LVEF) was 54%, 26.64% of patients had type 2 diabetes, and 28.70 had kidney failure. The average number of points, according to the Carlson comorbidity index, was four (Table 1).

Patients undergoing TLE for infectious reasons were older and more often male, and had a lower left ventricular ejection fraction (LVEF) and a higher incidence of comorbidities. Comparison of laboratory parameters showed a significantly higher level of leukocytes and neutrophils with lower lymphocyte values (along with higher NLR and LPR) in patients with CIED-related infections, when compared with the non-infectious group. The level of platelets was comparable in both study groups. Significantly higher levels of ESR and CRP were found in infectious patients with comparable procalcitonin values (Table 2).

**Figure 2:**
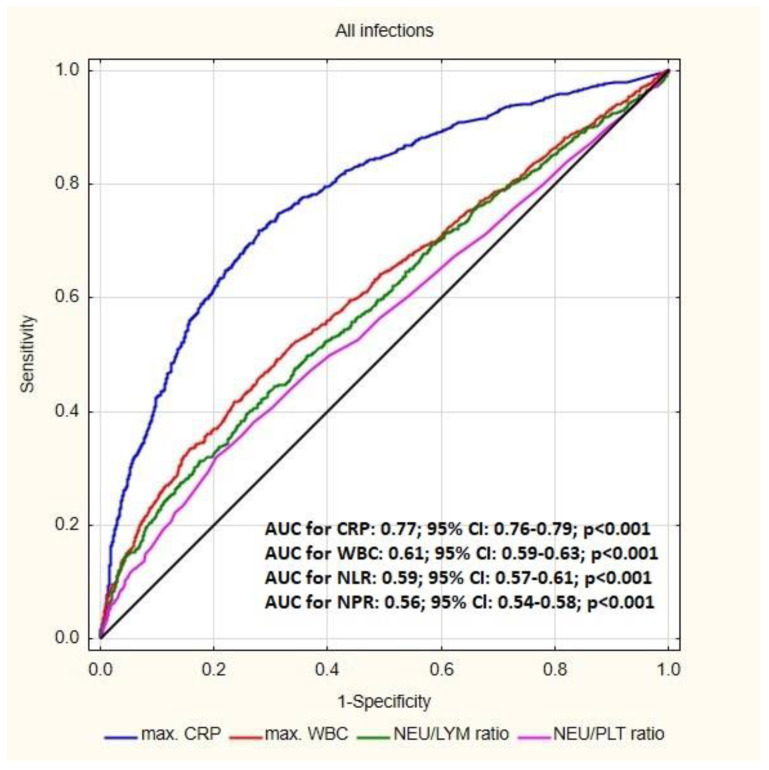
Receiver operating characteristic (ROC) curve for the blood parameters according to the presence of infectious complications in patients with cardiac implantable electronic devices (CIED), Abbreviations: AUC—area under curve, CRP—C-reactive protein, NLR—neutrophil-to-lymphocyte ratio, NPR—neutrophil-to-platelet ratio.

The receiver operating characteristic (ROC) curve analysis for infectious process in patients with CIED showed the highest area under curve (AUC) for CRP (0.77; *p* < 0.001) followed by WBC (0.61; *p* < 0.001), NLR (0.59; *p* < 0.001) and NPR (0.56; *p* < 0.001). The sensitivity and specificity for CRP was 72.66% and 70.62%; for WBC: 42.73% and 74.86%; for NLR: 40.54% and 72.82%; for NPR: 31.98% and 79.47% respectively. The optimal cut-off value in prediction of CIED-related infection was 3.06 for NLR and 0.02 for NPR (Figure 2).

Comparison of hematological parameters in patients (in whom the presence of additional intracardiac structures was demonstrated) referred to TLE for non-infectious and infectious reasons showed significantly higher NLR (3.37 vs. 2.61), NLR% (3.39 vs. 2.61), NPR (0.03 vs. 0.02), ESR (30.00 vs. 10.50 mm/h), and CRP (32.58 vs. 3.00 mg/L) parameters in patients with vegetation, compared with the group with vegetation-like masses (Table 3).

**Figure 3:**
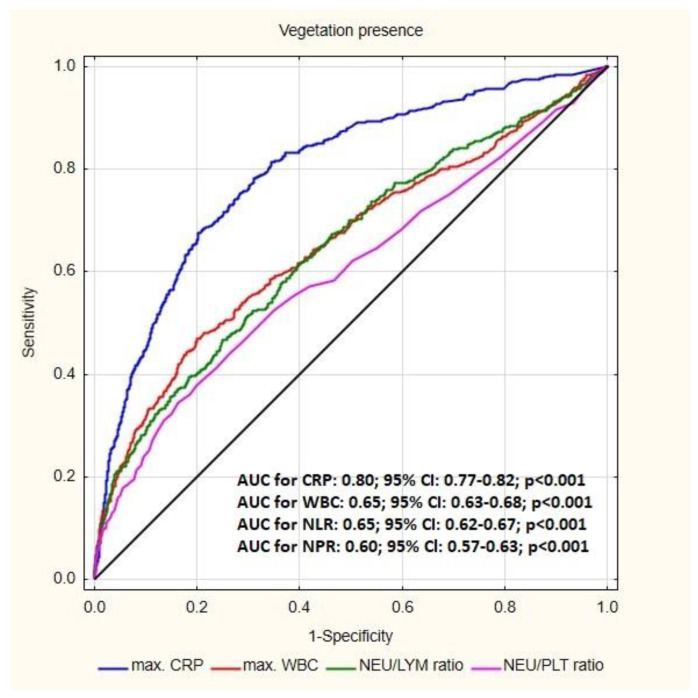
Receiver operating characteristic (ROC) curve for the blood parameters according to the presence of vegetation; Abbreviations: AUC—area under curve, CRP—C-reactive protein; NLR—neutrophil-to-lymphocyte ratio, NPR—neutrophil-to-platelet ratio.

The areas under the curve of the CRP, WBC, NLR and NPR for the prediction of vegetation was: 0.80 (*p* < 0.01), 0.65 (*p* < 0.01), 0.65 (*p* < 0.01) and 0.60 (*p* < 0.01) respectively. The highest sensitivity was shown for CRP (79.71%), and the highest specificity was found for NPR (82.78%). The optimal cut-off value for NPR in prediction of vegetations was 0.03 (Figure 3).

Comparison of the hematological markers in patients with LRIE and isolated pocket infection showed significantly higher NLR (4.11 vs. 2.55) and NLR% (4.24 vs. 2.56), higher NPR (0.03 vs. 0.02), lower LPR% (0.08 vs. 0.12), and higher standard inflammatory markers–maximal CRP (65.00 vs. 7.30 mg/L), ESR (45.50 vs. 15.00 mm/h), and procalcitonin (0.23 vs. 0.08) in the LRIE group (Table 4).

**Figure 4:**
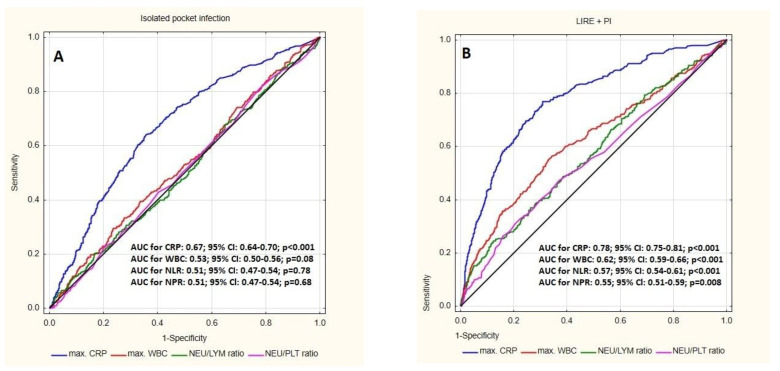
(**A**) Receiver operating characteristic (ROC) curve for the blood parameters according to the presence of isolated pocket infections (PI); (**B**) Receiver operating characteristic (ROC) curve for the blood parameters according to the coexistence of pocket infection (PI) with lead-related infection endocarditis (LRIE); Abbreviations: AUC—area under curve, CRP—C-reactive protein, WBC—white blood cells; NLR—neutrophil-to-lymphocyte ratio, NPR—neutrophil-to-platelet ratio.

The results of the ROC curve analysis for isolated pocket infection showed the highest AUC for CRP (0.67; *p* < 0.001) and lower AUC for WBC, NLR and NPR (respectively: 0.53; *p* = 0.08, 0.51; *p* = 0.78 and 0.51; *p* = 0.68). The sensitivity and specificity of CRP was 64.45% and 63.13% (Figure 4A).

The ROC curve analysis for coexistence LRIE with PI showed higher AUC for all analyzed parameters compared with isolated PI: CRP-0.78; *p* < 0.001, WBC-0.62; *p* < 0.001, NLR-0.57; *p* < 0.001 and NPR-0.55; *p* = 0.008. The CRP value was characterized by the highest sensitivity in the detection of coexistence LRIE with PI-77.49%; the highest specificity was demonstrated for NLR-87.33%. The optimal cut-off value for NLR in prediction of LRIE was 3.13 (Figure 4B).

## 4. Discussion

Hematological parameters, including novel markers such as neutrophil-to-lymphocyte ratio or platelet-to-lymphocyte ratio, appear to have a growing significance in the diagnosis and prognosis of diseases in many branches of medicine [13,14,15,16]. Currently, more and more studies suggest that NLR and NPR are inexpensive and easily accessible markers of inflammation in various infectious processes [17,18,19] and cardiovascular diseases [20,21]. The present study assessed the usefulness of the new laboratory parameters in the initial diagnosis of infection in patients with cardiac implantable electronic devices. In addition to an increase in typical inflammatory parameters (leucocytes, CRP, ESR and procalcitonin), comparative analysis of clinical data of patients referred for transvenous lead extraction procedures showed significantly higher NLR and NPR markers in patients with infectious indications for TLE. This study demonstrated the high sensitivity of CRP (72.66%) in the diagnosis of CIED infection; however, NLR and NPR were characterized by higher specificity compared to typical inflammatory parameters (79.47% for NPR and 72.82% for NLR vs. 70.62% for CRP). Moreover, this study documented that the optimal cut-off value in the prediction of infectious complications in patients with CIED is 3.06 for NLR and 0.02 for NPR.

The assessment of simple hematological parameters may also be helpful in the initial differentiation of additional structures associated with lead or surrounding tissues. In our study population, the presence of vegetation-like masses was observed in 4.02% of non-infectious patients while, according to the literature, additional lead-related structures were found in up to 5–28% of asymptomatic patients with CIED [22,23,24,25]. Histopathological analysis of the removed lead confirmed the frequent occurrence of connective tissue growths and thrombi in patients without symptoms of infection as a reaction to the presence of a foreign body [26,27,28]. Due to the possibility of bacterial colonization, it is very important to exclude an active inflammatory process in patients with lead-related structures. Naturally, advanced imaging techniques, including l8-Fluorodeoxyglucose PET/computed tomography (FDG-PET/CT) and 99mTc-hexamethypropylene-amine oxime labelled autologous white blood cell scintigraphy (WBC SPECT), can be very helpful in detecting inflammation, but they are associated with high costs [29,30,31]. Meanwhile, the comparative analysis of hematological markers in patients with vegetations and non-infectious patients with the presence of vegetation-like masses, conducted in the current study, showed increased typical inflammatory parameters in patients with vegetations. The highest sensitivity in detecting vegetation was demonstrated for CRP (79.71%), but the highest specificity was found for NPR (82.78%), with an optimal cut-off value of 0.03. 

The most dangerous form of CIED-related infection is lead-related infective endocarditis. LRIE may develop in patients with pockets of infection or occur in an isolated form. In patients with PI, there are often problems with assessing the extent of the infection, which may spread along the lead to the endocardium. As mentioned above, FDG-PET/CT and WBC SPECT imaging techniques can be very helpful in LRIE diagnosis; however, the use of this technique is still limited, and the need for cheap, non-invasive inflammatory parameters is increasing. The previous literature has not analyzed the usefulness of hematological parameters in the diagnosis of LRIE. One of the few studies documented that NLR could be considered a novel marker of bacteremia and/or sepsis. This study demonstrated the high sensitivity (57.8%) and specificity (83.9%) of this parameter in patients with bacteremia compared to the local infectious process [31]. Another study showed the highest predictive value of NLR in the detection of infective endocarditis: AUC under the ROC curve of NLR was 0.82; *p* < 0.001. Moreover, the high sensitivity and specificity of the parameter was demonstrated to be 69% and 88% [32]. High NLR may also predict the outcome of infective endocarditis, especially with the identified bacterial pathogen [8,32,33]. However, the above-mentioned studies do not focus on LRIE. In the present study, NLR, and NPR, together with standard markers (CRP, procalcitonin), were significantly higher in patients with LRIE compared with isolated PI group, which is essential information in a diagnostic process. Among the tested parameters, the highest sensitivity in assessing the spread of the infectious process from the pocket to the endocardium was demonstrated for CRP (77.49%), but the highest specificity in predicting LRIE was found for NLR (87.33%), with an optimal cut-off value of 3.13. These results may be important in the diagnosis of lead-related infective endocarditis and contribute to further insightful diagnostics and therapy.

## 5. Limitations

The main limitation of the study is its retrospective nature. Additionally, in the current study, a specific population of patients qualified for TLE was analyzed. In order to confirm the value of new hematological parameters, a comparative analysis of laboratory tests in patients, before and after CIED implantation, would also be advisable. Another limitation is the very rare use of new imaging methods, namely FDG-PET/CT and WBC SPECT, in the study population, which makes it impossible to compare the sensitivity and specificity of morphological parameters based on these techniques. 

## 6. Conclusions

Neutrophil-to-lymphocyte ratio and neutrophil-to-platelet ratio are novel and cheap markers that are useful in clinical practice in the diagnosis of the inflammatory process in patients with cardiac implantable electronic devices. NLR and NPR are characterized by high specificity in the initial diagnosis of CIED infections with the optimal cut-off 3.06 for NLR and 0.02 for NPR. NLR may also be helpful in a preliminary assessment of the spread of infection in patients with PI with the optimal cut-off 3.13. NPR is also useful in the differentiation of vegetations and vegetation-like masses on the leads, with the optimal cut-off value 0.03. 

## Figures and Tables

**Table 1:** Characteristics of study group.

	All Patients *n* = 2909
Patient’s age during TLE [years] median (Q1–Q3)	69 (59–77)
Patient’s age during first system implantation [years] median (Q1–Q3)	61 (51–69)
Female (*n*, %)	1340 (46.06)
LVEF median (Q1–Q3)	54 (36.00–60.00)
Renal failure (any) (*n*, %)	885 (28.70)
Diabetes type 2 (*n*, %)	775 (26.64)
Carlson’s comorbidity index [number of points] median, (Q1–Q3)	4.00 (2.00–6.00)
Infectious indications for TLE (*n*, %)	1029 (36.37)
Isolated pocket infections (*n*, %)	296 (10.18)
LRIE (with and without pocket infection) (*n*, %)	733 (25.20)
Positive blood cultures (*n*, %)	273 (37.24%)
Vegetations (*n*, %)	507 (17.40)
Non-infectious indications for TLE (*n*, %)	1880 (64.63)
Vegetations-like masses (*n*, %)	117 (4.02)

LRIE—lead related infective endocarditis, LVEF—left ventricle ejection fraction, TLE—transvenous lead extraction.

**Table 2:** Comparison of clinical and laboratory parameters in patients qualified for TLE for infectious and non-infectious causes.

	Infectious Indications for TLE *n* = 1029	Non-Infectious Indications for TLE *n* = 1880	*p*
Patient’s age during TLE [years] median (Q1–Q3)	70 (61–78)	68 (58–76)	<0.001
Patient’s age during first system implantation [years] median (Q1–Q3)	63 (54–71)	60 (48–68)	<0.001
Sex (% of female patients) (*n*, %)	306 (29.74)	828 (44.04)	0.001
LVEF [%] median (Q1–Q3)	50 (36.60)	55 (35.–60.)	<0.001
Renal failure (any) (*n*, %)	271 (26.34)	352 (18.72)	<0.001
Diabetes type 2 (*n*, %)	232 (22.55)	332 (17.66)	<0.001
Carlson’s comorbidity index [number of points] median, (Q1–Q3)	4.00 (3.00–7.00)	4.00 (2.00–5.50)	<0.001
Hemoglobin (g/dL) (lowest) (mean, SD)	12.5 (11.0–13.30)	13.30 (12.10–14.40)	<0.001
Hematocrit (%) (lowest) median (Q1–Q3)	37.20 (33.00–40.90)	39.90 (36.20–42.90)	<0.001
Platelets/µL (lowest) median Q1–Q3)	210.0 (164.0–272.0)	197.0 (160.0–241.0)	0.420
Max WBC/µL (mean.SD)	8185 (6600–10360)	7210 (6070–8630)	0.032
Neutrophil count/uL (max) median (Q1–Q3)	5.29 (3.90–7.40)	4.30 (3.50–5.50)	0.018
Neutrophil% median (Q1–Q3)	66.15 (58.60–74.00)	62.90 (56.60–69.10)	0.002
Lymphocyte count/µL (max) median (Q1–Q3)	1.60 (1.30–2.30)	1.70 (1.30–2.19)	<0.001
Lymphocyte% median (Q1–Q3)	22.30 (16.20–29.10)	24.60 (19.20–30.50)	<0.001
Max ESR (mm/h) median (Q1–Q3)	25.00 (1.00–50.00)	11.00 (6.00–20.00)	<0.001
Max CRP (mg/dL) median (Q1–Q3)	17.57 (5.07–60.40)	2.00 (0.60–7.17)	<0.001
Max Procalcitonin (µg/L) median (Q1–Q3)	0.10 (0.06–0.30)	0.07 (0.04–0.125)	0.154
NLR median (Q1–Q3)	3.07 (2.12–4.91)	2.59 (1.86–3.57)	<0.001
NLR% median (Q1–Q3)	3.07 (2.12–4.84)	2.57 (1.86–3.58)	<0.001
NPR median (Q1–Q3)	0.02 (0.02–0.04)	0.01 (0.01–0.03)	0.008
LPR% median (Q1–Q3)	0.10 (0.07–0.15)	0.13 (0.10–0.17)	0.001
LPR (median IQR)	0.01 (0.01–0.01)	0.01 (0.01–0.01)	0.003

Abbreviations: CRP—C-reactive protein, ESR—Erythrocyte Sedimentation Rate, LPR—lymphocyte-to-platelet ratio, NLR—neutrophil-to-lymphocyte ratio, NPR—neutrophil-to-platelet ratio, WBC—white blood cells.

**Table 3:** Comparison of hematological parameters of patients with vegetation-like masses and patients with vegetation.

Parameters	Presence of Vegetation-Like Masses	Presence of Vegetations	*p*
Hemoglobin (mg/dL) (lowest) median (Q1–Q3)	13.70 (11.90–14.70)	11.90 (10.30–13.20)	<0.001
Hematocrit (%) (lowest) median (Q1–Q3)	40.20 (36.00–43.90)	35.90 (31.20–39.70)	<0.001
Platelets/µL (lowest) median (Q1–Q3)	200.0 (163.0–252.0)	215.0 (157.0–278.0)	0.063
Max WBC/µL median (Q1–Q3)	7440 (6060–8640)	8880 (7000–11,400)	<0.001
Neutrophil count/µL (max) median (Q1–Q3)	4.50 (3.56–5.54)	5.70 (4.10–8.30)	<0.001
Neutrophil% median (Q1–Q3)	63.40 (54.80–69.80)	67.70 (60.40–76.20)	<0.001
Lymphocyte count/µL (max) median (Q1–Q3)	1.65 (1.38–2.10)	1.60 (1.20–2.20)	0.927
Lymphocyte% median (Q1–Q3)	24.30 (18.70–31.90)	19.80 (13.60–26.10)	<0.001
Max ESR (mm/h) median (Q1–Q3)	10.50 (5.00–19.50)	30.00 (13.00–54.00)	<0.001
Max CRP (mg/dL) median (Q1–Q3)	3.00 (0.67–10.75)	32.58 (9.30–90.00)	<0.001
Max Procalcitonin (µg/L) median (Q1–Q3)	0.05 (0.04–1.52)	0.12(0.07–0.50)	0.581
NLR median (Q1–Q3)	2.61 (1.72–3.67)	3.37 (2.35–5.55)	<0.001
NLR% median (Q1–Q3)	2.61 (1.70–3.81)	3.39 (2.37–5.56)	<0.001
NPR median (Q1–Q3)	0.02 (0.02–0.03)	0.03 (0.02–0.04)	0.008
LPR% median (Q1–Q3)	0.13 (0.09–0.18)	0.09 (0.06–0.14)	0.025
LPR median (Q1–Q3)	0.01(0.01–0.01)	0.01 (0.01–0.01)	0.937

Abbreviations: CRP—C-reactive protein, LPR—lymphocyte-to-platelet ratio; NLR—neutrophil-to-lymphocyte ratio, NPR—neutrophil-to-platelet ratio, WBC—white blood cells.

**Table 4:** Hematological parameters of patients undergoing TLE due to LRIE and isolated PI.

Parameters	LRIE	Isolated Pocket Infection	*p*
Hemoglobin (g/dL) (lowest) median (Q1–Q3)	11.90 (10.50–13.30)	13.20 (12.00–14.30)	<0.001
Hematocrit (%) (lowest) median (Q1–Q3)	33.95 (29.15–37.80)	39.30 (35.90–42.10)	<0.001
Platelets/µL (lowest) median (Q1–Q3)	222.0 (159.0–293.0)	202.00 (166.0–250.5)	0.001
Max WBC/µL (mean.SD)	10,100 (7550–13,300)	7370 (6280–8900)	<0.001
Neutrophil count/µL (max) median (Q1–Q3)	6.80 (4.65–9.50)	4.40 (3.60–5.80)	<0.001
Neutrophil % median (Q1–Q3)	71.10 (63.75–78.45)	62.75 (56.25–70.50)	<0.001
Lymphocyte count/µL (max) median (Q1–Q3)	1.60 (1.02–2.2.0)	1.70 (1.30–2.29)	0.93
Lymphocyte% median (Q1–Q3)	17.00(11.45–23.30)	24.70 (18.80–30.30)	<0.001
Max ESR (mm/h)median (Q1–Q3)	44.50 (22.00–68.00)	15.00 (8.00–30.00)	<0.001
Max CRP (mg/dL) median (Q1–Q3)	65.00 (24.70–120.7)	7.30 (2.20–19.20)	<0.001
Max Procalcitonin (µg/L) median (Q1–Q3)	0.23 (0.10–1.53)	0.08 (0.05–0.10)	0.03
NLR median (Q1–Q3)	4.11 (2.72–6.98)	2.55 (1.85–3.70)	<0.001
NLR% median (Q1–Q3)	4.24 (2.75–6.93)	2.56 (1.87–3.73)	<0.001
NPR median (Q1–Q3)	0.03 (0.02–0.05)	0.02 (0.02–0.03)	<0.001
LPR% median (Q1–Q3)	0.08 (0.05–0.11)	0.12 (0.08–0.16)	0.001
LPR median (Q1–Q3)	0.01 (0.00–0.01)	0.01 (0.01–0.01)	0.41

Abbreviations: CRP—C-reactive protein, ESR—Erythrocyte Sedimentation Rate, LPR—lymphocyte-to-platelet ratio; LRIE—lead related infective endocarditis, NLR—neutrophil-to-lymphocyte ratio, NPR—neutrophil-to-platelet ratio, WBC—white blood cells.

## Data Availability

The corresponding author has complete access to the original study data, and anonymized data will be made available upon request with a reasonable purpose.
